# Configural analysis of dry-land strength and front crawl performance in adolescents

**DOI:** 10.3389/fphys.2025.1631224

**Published:** 2025-09-17

**Authors:** Tianyi Gao, Jiawen Shen, Baojie Tang, Xinyuan Wu, Yifan Shi, Bo Huang

**Affiliations:** ^1^ School of Sports Science, South China Normal University, Guangzhou, Guangdong, China; ^2^ The Affiliated High School of South China Normal University, Guangzhou, Guangdong, China; ^3^ Graduate School, Guangzhou Institute of Physical Education, Guangzhou, Guangdong, China

**Keywords:** configural analysis, QCA, front crawl swimming performance, dry-land strength, adolescent athletes

## Abstract

**Introduction:**

Dry-land strength capacities play a crucial role in competitive swimming, especially in short-distance events where explosive force and coordination are decisive. However, most research has focused on isolated variables rather than exploring how combinations of strength attributes jointly influence performance. Competitive swimming performance is influenced by multiple interacting physical attributes, yet the specific combinations of dry-land strength capacities that contribute to short-distance front crawl performance in adolescents remain unclear.

**Methods:**

To address this gap, this study employs fuzzy-set Qualitative Comparative Analysis (fsQCA) to investigate the configurational relationships between dry-land strength parameters and 50-m front crawl swimming performance among adolescent competitive swimmers. Eighty-five adolescent competitive swimmers (n = 85; age: 15.0 ± 1.5 years; weight: 61.5 ± 9.6 kg) were categorized into three groups based on competition scores and underwent seven physical assessments, including deep squats, pull-ups, grip strength tests, medicine ball throws, progressive plank, and vertical jumps.

**Results:**

Using fsQCA 3.0 software, configuration analysis revealed six significant causal configurations explaining 72.7% of high-performance cases. Configurations S1a/S1b identified core conditions in deep squats, pull-ups, and grip strength, while S2a/S2b highlighted bench press and vertical jumps for enhancing stroke efficiency and start/turn acceleration. The S3/S4 configurations demonstrated unique contributions from whole-body coordination and vertical explosiveness, respectively.

**Discussion:**

Multifactor synergy is key to improving swimming performance, and different athletes may need an individualized training focus. Coaches should develop a training plan based on the specific needs of the athletes to maximize their potential.

## 1 Introduction

Contemporary competitive swimming performance is influenced by multiple dry-land strength attributes, including upper- and lower-limb power and core stability. Although several studies have investigated the effects of isolated strength components on swimming performance, there is a lack of research examining how different strength qualities interact in combination to influence outcomes, particularly in adolescent swimmers. This gap is critical, as performance at the youth level may depend more on synergistic physical profiles than isolated capabilities. Previous investigations have demonstrated that structured dry-land strength interventions over several weeks elicit positive adaptations in key swimming performance metrics, including stroke efficiency and propulsive force generation (Amaro et al., 2017; Lopes et al., 2021; Sadowski et al., 2012). Upper limb muscular strength has consistently shown significant correlations with competitive swimming outcomes (Pérez-Olea et al., 2018). Elite athletes have demonstrated a superior ability to transfer upper-body strength and power into aquatic propulsion (Tan et al., 2024). As a result, current training practices continue to prioritize upper-body strength development as a key component of athletic preparation (Crowley et al., 2018). Likewise, core strength training is considered an essential part of comprehensive swim training programs, as it positively influences technical aspects such as start performance, turn execution, and stroke mechanics (Lopes et al., 2021; Karpiński et al., 2020; Crowley et al., 2017). Lower-extremity strength development has also remained a consistent focus in dry-land programming (Sammoud et al., 2021), with studies reporting benefits in maximal leg strength, kicking efficiency, turn performance, and overall race outcomes (Amara et al., 2022; Jones et al., 2018).

Dry-land strength tests commonly employed in swimming research are designed to reflect the biomechanical demands of front crawl propulsion, start, and turn phases. These tests are not arbitrary; rather, they target specific muscle groups known to contribute to propulsion and stroke efficiency. For instance, pull-up exercises primarily engage the latissimus dorsi and biceps brachii, key contributors to force production during the underwater pull phase of the stroke (Garrido et al., 2010; McLeod, 2010). Complementary pushing movements such as the bench press and medicine ball throw activate the pectoralis major and triceps brachii, supporting anterior propulsion during arm extension (McLeod, 2010). Lower-limb strength, typically assessed through squats and vertical jumps, involves the quadriceps and gluteal muscles, which play a central role in explosive actions during starts and wall turns (Garrido et al., 2010; McLeod, 2010). In addition, core stability, evaluated through variations of the plank test, is essential for maintaining streamlined alignment and enabling coordinated force transfer across the kinetic chain, ultimately enhancing stroke efficiency and minimizing drag (McLeod, 2010). By grounding test selection in these biomechanical principles, we ensure functional relevance between the strength measures assessed and the swimming performance outcomes studied. This rationale supports our use of a configurational approach to identify how combinations of these strength traits relate to competitive performance.

Dry-land strength parameters have been established as primary predictors of competitive swimming performance in prior research (Martens et al., 2015). The pull-up exercise predominantly engages the latissimus dorsi - a prime mover in front crawl propulsion exhibiting substantial electromyographic activation during swimming. Empirical evidence identifies pull-up capacity as a robust predictor of swimming performance metrics (Pérez-Olea et al., 2018). Maximal handgrip strength, primarily dependent on forearm flexors (flexor carpi radialis and digitorum superficialis), demonstrates significant correlations with sprint front crawl performance. Beyond localized adaptations, grip training enhances upper limb kinetic chain control and force transfer efficiency (Garrido et al., 2012; Geladas et al., 2005). The bench press exercise recruits the pectoralis major as the primary agonist, with secondary contributions from triceps brachii and anterior deltoids, while rotator cuff musculature maintains glenohumeral stability (Stastny et al., 2017). Maximal strength measures, including 1RM bench press, back squat, and vertical jump height, show strong associations with sprint swimming performance, particularly in distances ≤100 m where maximal force production predominates (Keiner et al., 2021). Medicine ball chest throws effectively develop upper body power through triple extension mechanics: pectoralis major drives anterior humeral translation, anterior deltoids assist in frontal plane movement, and triceps brachii facilitates elbow extension. Improvements in throwing distance correlate with enhanced sprint swimming velocity (Lopes et al., 2021). Core musculature is operationally defined as the myofascial structures between the sternum and knees, emphasizing abdominal (rectus abdominis, transversus abdominis), lumbar (erector spinae), and pelvic (gluteus maximus/medius) regions (Tong et al., 2014). The Progressive Plank test serves as a validated assessment tool for dry-land core stability in athletic populations (Ruijie et al., 2016).

The implementation of athlete-specific training protocols in competitive swimming demonstrates substantial variability across performance levels. Coaching practitioners typically prescribe individualized training programs guided by comprehensive assessments of athletes’ unique physiological and biomechanical profiles, particularly when working with elite competitors (Yu-hong and Mai-jiu, 2009). However, emerging evidence reveals significant challenges in applying true personalization principles to adolescent swimming populations. This operational reality is reflected in the current paucity of high-quality empirical studies examining case-specific training interventions in aquatic sports, particularly within the adolescent cohort. Given the increasing interest in individualized strength assessment and the complex interplay of strength variables in youth athletes, this study explores how combinations of dry-land strength metrics relate to 50-m freestyle performance. Rather than aiming to isolate single predictors, we adopt a configurational perspective using fuzzy-set Qualitative Comparative Analysis (fsQCA), a methodological approach that identifies combinations of conditions (configurations) associated with a specific outcome, particularly useful for exploring causal complexity in small to medium samples (Rihoux and Ragin, 2009; Wang et al., 2022). Coaches may leverage these findings to design targeted interventions addressing individualized force-velocity curve optimization and intermuscular coordination patterns inherent to high-velocity swimming.

Previous studies have highlighted that elite swimmers achieve superior propulsion by combining strong upper-body pulling actions (e.g., latissimus dorsi and pectoral engagement) with powerful lower-body pushing mechanics during starts and turns (Pérez-Olea et al., 2018; Tan et al., 2024; Sammoud et al., 2021; Amara et al., 2022; Jones et al., 2018). This suggests that swimming performance is not driven by a single strength attribute, but rather by the coordinated synergy of upper-limb, core, and lower-limb power outputs. Building on this evidence, we hypothesize that specific combinations of dry-land strength variables, particularly upper-pull and lower-push capacities, together with core stability, are key determinants of short-distance front crawl performance in adolescent athletes. To evaluate these interactions, we applied fsQCA, a method particularly suited to uncover complex, non-linear configurations of conditions that collectively lead to high performance, which traditional linear models may fail to capture.

## 2 Methods

### 2.1 Participants

This study employed a stratified sampling method to recruit 85 adolescent competitive swimmers based on the World Aquatics Point Scoring System 2025 for the 50 m freestyle event. This system is internationally recognized for standardizing performance across age and gender categories and was used here to classify swimmers into performance-based groups. Group I: 23 athletes with ≥621 points. Group II: 42 athletes scoring 440–620 points, indicative of intermediate-level performance typically seen in regional or national developmental athletes. Group III: 20 athletes with <440 points, reflecting early-stage or lower-performing competitive athletes. The full anthropometric and performance characteristics are detailed in Table 1. Although all participants were national-level swimmers, the groups differed in average age and training history (e.g., Group I: 16.2 years, 9.0 years of training vs. Group III: 14.0 years, 4.5 years). This classification approach is consistent with prior literature using FINA-based stratification to assess swimmer development and performance (Crowley et al., 2017; Keiner et al., 2021). Sex distribution and pubertal maturation status were not recorded, which may contribute to unmeasured heterogeneity in physical development and training responsiveness. Grouping was based solely on performance level (FINA points), without adjustment for maturational stage or sex. All participants underwent comprehensive protocol briefings before providing written informed consent. The study protocol was approved by the Science Research Ethics Sub-Committee of the School of Sports Science, South China Normal University (Approval No. SCNU-SPT-2025-012).

**TABLE 1:** Basic information of the athletes.

Group	Point	Number	Age	Years	Height	Weight
1	≥621	23	16.2 ± 1.2	9.0 ± 2.3	180.2 ± 6.1	69.5 ± 7.9
2	620–440	42	14.6 ± 1.5	7.6 ± 2.4	168.4 ± 8.1	57.3 ± 9.1
3	<440	20	14.0 ± 1.2	4.5 ± 1.6	168.0 ± 10.7	58.1 ± 9.8

### 2.2 Procedure

Strength capacities can be classified through biomechanical, physiological, and anatomical perspectives. For comprehensive evaluation of adolescent short-course front crawl swimmers, this study adopts an anatomical framework dividing strength capacities into Upper Limb Strength, Core Stability, and Lower Limb Strength. The investigation employed a biphasic testing framework comprising seven land-based physiological assessments administered consecutively with standardized 5-min active recovery intervals, followed by aquatic performance evaluation 24 h post-terrestrial testing.

### 2.3 Terrestrial evaluation phase

#### 2.3.1 Warm-up protocol

All participants must complete: (i) Dynamic muscle activation sequence (8–10 joint-specific movements); (ii) Sport-specific energy system preparation (RPE 12–14 on Borg 6–20 scale).

#### 2.3.2 Bench press 1RM testing

Start with a weight that can be comfortably lifted 8–10 times as a warm-up, then rest for 1 min. Increase the initial load by 5%–10% and begin the bench press test. Repeat this process until you can no longer lift the weight 3–5 times. Use the one repetition maximum (1RM) calculation formula: 1RM = Test weight × Bench press coefficient to calculate the tester’s 1RM bench press weight (Haff and Triplett, 2015) (Table 2). Previous studies have shown that bench press strength contributes to stroke propulsion, particularly through activation of the pectoralis major and triceps during the pull phase (Tan et al., 2024; Stastny et al., 2017; Yu Kwok et al., 2021).

**TABLE 2:** Table of corresponding coefficient of one repetition maximum in bench press.

Maximum number of replicates	Bench press coefficient
1	1.000
2	1.035
3	1.080
4	1.115
5	1.150
6	1.180
7	1.220
8	1.255
9	1.290
10	1.325

#### 2.3.3 Handgrip dynamometry

Adjust the Jamar dynamometer to the 2nd handle position. (1) Seated protocol: Shoulder adducted, elbow 90° flexion. Three maximal contractions per hand (60s inter-trial recovery). Outcome Measure: Mean peak force (kg) from best trial per limb. Grip strength is associated with better upper-limb force transmission and control during front crawl, and correlates with sprint performance (Garrido et al., 2012; Geladas et al., 2005).

#### 2.3.4 Seated medicine ball throw

Experimental Setup: (1) Standardized bench: 45° backrest with pelvic stabilization strap; (2) Implementations: Medical balls (3 kg); (3) Testing Sequence: After the throw, the staff measured and recorded the distance between the starting point and the landing point, and the result was accurate to 1 cm. Take two tests and rest and recover for 2–3 min after each test, taking the best score record. Medicine ball throws have been linked to improvements in explosive upper-body power and swimming sprint velocity (2).

#### 2.3.5 Strict pull-up assessment

Prerequisites: (1) Scapular mobility screening (Kirby test ≥160°). (2) Pre-test activation: 2 × 10 band-assisted reps. Technical Execution Standards: Bottom: Elbow extension ≤175° (goniometric verification); Top: Chin clear horizontal plane of bar; Disallowed mechanics: Kipping/bouncing movements; Asymmetrical pulling patterns. Pull-up performance has been identified as a key indicator of latissimus dorsi strength, directly impacting underwater pulling effectiveness (Pérez-Olea et al., 2018; Pardo-Atarés et al., 2024).

#### 2.3.6 Progressive plank

This test involves eight progressively challenging levels of abdominal bridge exercises, each level increasing in difficulty (Table 3). Starting from the easiest position, gradually move to more difficult positions according to the instructions. The subject should maintain each level for a specified amount of time or until failure. Record the level achieved and the duration maintained at that level before the subject can no longer hold the position properly. The cumulative score quantifies the athlete’s ability to maintain postural control under increasing asymmetrical and unstable loading, providing a functional measure of core stability. Core stability is critical for hydrodynamic positioning and efficient force transfer during swimming. The progressive plank has shown validity as a core strength assessment for swimmers (Ruijie et al., 2016; Yu-hong and Mai-jiu, 2009).

**TABLE 3:** Progressive plank assessment scale.

Stage	Biomechanical requirements	Temporal standard	Tier score	Cumulative score
I	Bilateral forearm/plantar support	60s	1	1
II	Left unilateral forearm/bipedal stabilization	15s	3	4
III	Right unilateral forearm/bipedal control	15s	5	9
IV	Bilateral forelimb/left unipedal load-bearing	15s	6	15
V	Bilateral forelimb/right unipedal static demand	15s	10	25
VI	Cross-limb stability (left forearm/right pedal)	15s	15	40
VII	Cross-limb stability (right forearm/left pedal)	15s	25	65
VIII	Bilateral forearm/plantar support	30s	35	100

#### 2.3.7 Vertical jump assessment

Execution Standards Initial posture: Hip-width stance with hands maintained on iliac crests. (1) Countermovement depth: 30° knee flexion Take-off/landing requirements: Maintain sagittal plane alignment. Measure and record the height jumped from the starting position to the peak of the jump. Each subject performs two attempts, with 2–3 min of rest between attempts, recording the best result. Vertical jump height reflects lower-limb explosive power, a key determinant of start and turn performance in sprint swimming (Keiner et al., 2021; Born et al., 2020; Zebura et al., 2023) and was measured using a force plate system, based on ground reaction force and flight time.

#### 2.3.8 Push-off back squat test

Begin with a thorough warm-up, including dynamic stretches focused on the lower body. Start with a light weight that allows for an easy completion of 8–10 repetitions as part of the warm-up. Increase the weight by 5%–10% and start the back squat test. Ensure proper form is maintained throughout the test: feet flat on the ground, knees aligned with toes, back straight, and descend until thighs are parallel to the floor. Repeat until you can no longer perform the movement correctly. Depending on the specific goal, this could be measured through repetitions until failure or by calculating a one-repetition maximum (1RM) like the bench press test. 1RM = test weight × back squat coefficient (Table 4). Back squat strength is linked to lower-limb power and contributes to faster starts and wall push-offs in competitive swimmers (Keiner et al., 2021; Zebura et al., 2023).

**TABLE 4:** Table of corresponding coefficient of one repetition maximum in back squats.

Maximum number of replicates	Back squat coefficient
1	1.0000
2	1.0475
3	1.1300
4	1.1575
5	1.2000
6	1.2420
7	1.2840
8	1.3260
9	1.3680
10	1.4100

### 2.4 Aquatic performance phase

Swimming performance was evaluated through a maximal-effort 50 m front crawl sprint. Each swimmer performed a continuous two-length swim (2 × 25 m) in a 25 m pool. Prior to testing, swimmers completed a standardized warm-up protocol: 10 min of dynamic land-based activation, 15 min of in-water preparation, and a 10-min rest period. The trials were conducted individually to ensure maximal effort and eliminate drafting or pacing effects, in line with previous protocols (Jones et al., 2018; Born et al., 2020). Swimmers performed a standard dive start from the platform, and time was recorded using handheld stopwatches by experienced personnel positioned at the finish wall. Although manual timing introduces a small margin of error, this method has been commonly used in similar studies with acceptable reliability (Keiner et al., 2021; Zebura et al., 2023). To control for effort and consistency, tests were repeated if the performance fell below 90% of the swimmer’s personal best or if a false start occurred. The best of two valid trials was retained for analysis, following protocols similar to those used by Tan et al. (Tan et al., 2024).

### 2.5 Statistical analyses

To examine the combinatorial effects of strength variables on swimming performance, we applied fsQCA, a method rooted in Boolean algebra and set theory. FsQCA identifies configurations of conditions that are sufficient or necessary for a specific outcome, making it well-suited for investigating complex, multi-causal phenomena such as sport performance (Wang et al., 2022). Unlike traditional regression-based approaches that assume linear, additive, and symmetric effects, fsQCA accommodates causal asymmetry and equifinality, acknowledging that athletes may reach similar performance levels through distinct combinations of physiological attributes. This approach has previously been used in athletics research, for example, in javelin throwing, where athletes with contrasting physical profiles achieved equivalent performance through different factor combinations (Wang et al., 2022). FsQCA does not require large samples for statistical inference, as it relies on set-theoretic logic rather than probabilistic assumptions. According to methodological guidelines (Ragin, 2009), small-to-medium-N studies, typically ranging from 10 to 100 cases, are considered appropriate and even ideal for generating meaningful configurational results. Our sample of 85 swimmers falls within this range, offering sufficient diversity to identify relevant condition combinations. This sample size is consistent with other recent fsQCA applications in sport performance contexts, such as Wang et al. (2022), who analyzed elite javelin throwers using a comparable number of cases. We applied a minimum raw consistency threshold of 0.80 and a PRI (Proportional Reduction in Inconsistency) threshold of 0.75, as recommended by Ragin (Ragin, 2009) and Schneider & Wagemann (Schneider and Wagemann, 2012). These thresholds are commonly used in medium-N studies and offer a balance between explanatory power and solution diversity. Although some variables (e.g., medicine ball throw) appeared less frequently in the final configurations, we retained them due to their established role in swimming literature as indicators of upper-body power. This helped ensure a comprehensive assessment of relevant strength domains.

The dataset comprising all performance metrics was systematically processed for configuration analysis using fsQCA 3.0 software to examine the complex causal relationships between land-based strength parameters and 50 m front crawl swimming performance, with the latter designated as the outcome variable and the former as conditional variables. Calibration procedures, which function to operationalize abstract concepts into quantifiable fuzzy-set membership scores, transformed both outcome and conditional variables into membership degrees ranging from 0 (full non-membership) to 1 (full membership). Given the absence of standardized empirical cut-offs in the strength–swimming performance domain, we adopted a percentile-based calibration approach following guidelines from Ragin (2009) and Schneider and Wagemann (2012). Specifically, we used the 75th percentile for full membership, 50th percentile (median) for the crossover point, and 25th percentile for full non-membership. This approach ensures sample-specific calibration without imposing arbitrary or externally mismatched thresholds. While no universal benchmarks exist for these strength measures in adolescent swimmers, this strategy is widely applied in small-to-medium-N fsQCA studies when theoretical thresholds are unavailable. Additionally, to assess the robustness of our findings, we performed sensitivity checks by adjusting calibration thresholds ±5% for selected variables. These adjustments yielded similar configurations and consistency scores, confirming the stability of the original results.

For calibration purposes only, additional swimming-specific strength metrics were collected using two instruments: (Amaro et al., 2017) a tethered-swimming ergometer to assess peak and mean power output during a 15-s all-out front crawl effort, and (Lopes et al., 2021) an underwater force mounted on the pool wall to measure wall push-off force during a standard start. These data were not included in the fsQCA models but were used to validate the dry-land strength measures through correlational comparisons (Table 5).

**TABLE 5:** The fuzzy set calibration and descriptive statistics of the variables.

Variable	Descriptive statistics			Calibrate the anchor point		
	Number	Average	Standard deviation	Full membership	Crossover point	Full non-membership
Bench Press	85	46.2	17.3	55	44.6	33.5
back squat	85	90.9	36.4	110	79.1	66
Pull-ups	85	10.9	10.1	17	9	3
Progressive Plank	85	0.6	0.5	1	1	0
Medicine Ball	85	256.7	55.3	293	247	217
Handgrip Dynamometry	85	35.0	10.6	42.1	34.1	25.6
Vertical Jump	85	49.6	12.5	60	48	41
Performance	85	29.9	3.4	27.3	29.1	31.4

## 3 Results

### 3.1 Necessity analysis

Prior to conducting configuration effect analysis, we performed a necessity examination of individual conditional variables through fsQCA 3.0 by computing consistency and coverage metrics. Consistency, measured on a 0–1 scale, quantifies the directional association strength between conditional variables and the outcome variable, with values approaching 1.0 indicating that the conditional variable’s presence systematically corresponds to outcome occurrence across cases. Coverage estimates the explanatory scope by evaluating the proportion of outcome-positive cases where the conditional variable is present. Diagnostic thresholds followed convention from comparative sports analytics: conditional variables exceeding 0.9 consistency threshold were considered potentially necessary, whereas values below this cutoff indicated requirement for synergistic multi-factor configurations. A value of less than 0.9 indicates that the variable explains the outcome variable together with other conditional variables. The calculation shows that each condition variable showed a certain degree of correlation in the cases of excellent sports performance, but the performance in the non-high-sports performance cases is not enough to establish the necessary condition. Therefore, it is necessary to explore the way to achieve excellent front crawl swimming results and to comprehensively investigate the multiple synergies of multiple factors (Table 6).

**TABLE 6:** Necessality tests for conditional variables.

Variables	Excellent performance		Non-excellent performance	
	Consistency	Coverage	Consistency	Coverage
Bench Press	0.829876	0.812528	0.289776	0.30005
∼Bench Press	0.285106	0.275142	0.818948	0.835821
back squat	0.813633	0.793203	0.301289	0.310631
∼back squat	0.292876	0.283847	0.799423	0.819378
Pull-ups	0.835371	0.824967	0.285244	0.297906
∼Pull-ups	0.289051	0.276617	0.832406	0.842453
Progressive Plank	0.892256	0.670182	0.415208	0.329818
∼Progressive Plank	0.107744	0.148367	0.584792	0.851633
Medicine Ball	0.69812	0.666636	0.418916	0.42305
∼Medicine Ball	0.395803	0.391753	0.669894	0.701208
Handgrip	0.794994	0.763395	0.330724	0.33586
∼Handgrip	0.308368	0.303461	0.767012	0.798256
Vertical Jump	0.748469	0.722565	0.343153	0.350346
∼Vertical Jump	0.327056	0.320105	0.728261	0.753814

### 3.2 Conditional configuration analysis

The analytical protocol established parameter thresholds with raw consistency at 0.80, PRI consistency at 0.75, and minimum case frequency of 1, incorporating the foundational assumption in counterfactual analysis that both presence and absence of individual land-based strength training modalities could contribute to elite freestyle performance outcomes. Core conditions were identified through the comparative nesting of intermediate and parsimonious solutions, defined as variables appearing in both solution types, while peripheral conditions existed solely in intermediate solutions. This methodological framework revealed six distinct causal configurations (Table 7), with overall solution consistency reaching 0.929, surpassing theoretical sufficiency thresholds. The aggregate coverage of 0.727 indicated that these multi-factor pathways explained 72.7% of high-performance cases.

**TABLE 7:** Configurations of freestyle swimming performance in qualitative comparative analysis.

Condition variables	Configurations					
	S1a	S1b	S2a	S2b	S3	S4
Bench Press	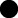	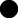	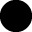	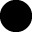	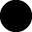	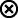
Back squat	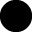	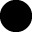	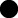		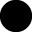	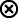
Pull-ups	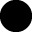	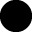	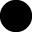	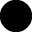		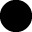
Progressive Plank	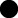		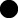	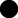	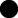	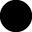
Medicine Ball				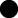	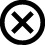	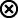
Handgrip Dynamometry	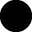	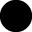		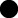	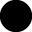	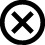
Vertical Jump		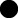	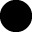	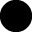	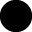	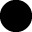
Raw Coverage	0.578	0.488	0.402	0.155	0.057	0.578
Unique Coverage	0.127	0.034	0.028	0.019	0.026	0.127
Consistency	0.953	0.946	0.933	0.967	0.897	0.953
Solution Coverage	0.727					
Solution Consistency	0.929					

● (large circle): Presence of a core condition variable.

● (small circle): Presence of a peripheral condition variable.

⊗ (large circle): Absence of a core condition variable.

⊗ (small circle): Absence of a peripheral condition variable.

Blank: Irrelevant or optional condition.

### 3.3 Robustness verification

The study conducted sensitivity checks on optimal freestyle performance configurations by recalibrating anchor points to 80%, 50%, and 20% percentiles. Core configurations demonstrated sustained robustness, with statistically insignificant variations in solution consistency and coverage metrics across threshold adjustments. A formal subset consistency analysis revealed hierarchical relationships among configuration patterns, satisfying necessity conditions for causal robustness, thereby validating the methodological dependability of conclusions (Table 8).

**TABLE 8:** Robustness test table.

Condition variables	Configurations					
	S1	S2	S3	S4	S5	S6
Bench Press	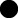	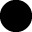	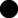	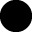	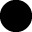	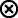
Back squat	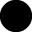	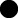	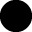	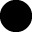		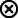
Pull-ups	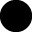	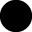	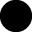		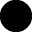	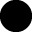
Progressive Plank	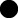	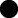		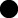	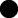	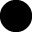
Medicine Ball				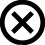	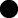	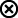
Handgrip Dynamometry	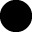		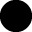	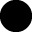	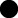	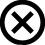
Vertical Jump		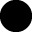	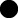	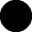	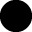	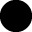
Raw Coverage	0.488	0.488	0.155	0.402	0.057	0.488
Unique Coverage	0.034	0.036	0.019	0.028	0.026	0.034
Consistency	0.946	0.949	0.967	0.933	0.897	0.946
Solution Coverage	0.727					
Solution Consistency	0.929					

● (large circle): Presence of a core condition variable.

● (small circle): Presence of a peripheral condition variable.

⊗ (large circle): Absence of a core condition variable.

⊗ (small circle): Absence of a peripheral condition variable.

Blank: Irrelevant or optional condition.

## 4 Discussion

The present findings align with recent investigations into dry-land strength application and its impact on sprint swimming performance (Tan et al., 2024; Pardo-Atarés et al., 2024; Zebura et al., 2023), reinforcing the relevance of updated training models and analytical frameworks for adolescent athletes.

Rather than treating each configuration in isolation, we highlight their key similarities and distinctions below to provide a clearer synthesis.

### 4.1 S1a and S1b

The S1a configuration is characterized by back squat, pull-ups, and grip strength as core condition variables, with bench press and eight-level core stabilization as peripheral factors, while medicine ball chest throws and vertical jump are non-essential. This combination reflects a strength profile focused on posterior chain engagement and trunk control. Similarly, the S1b configuration identifies back squat, pull-ups, and grip strength as foundational requirements, with bench press and vertical jump as peripheral contributors. Although the peripheral variables differ slightly, both S1a and S1b prioritize similar physical qualities. These configurations may support freestyle performance through complementary strength contributions at different race phases. For example, the back squat develops lower-extremity triple-extension power, which is relevant for block starts and turn thrusts, as shown by Zebura et al. (Zebura et al., 2023), who reported associations between lower-body maximal strength and start velocity in elite swimmers (Zebura et al., 2023). Pull-up strength reflects latissimus dorsi function and contributes to sprint performance and swimming speed (Pérez-Olea et al., 2018; Pardo-Atarés et al., 2024). Although grip strength remains less studied, it may contribute to stable hand positioning during propulsion. While our findings suggest a possible functional synergy among these strength components, we did not directly assess neuromuscular coordination or in-water force timing. Future research using EMG or kinetic analyses could clarify how dry-land strength qualities influence race-segment-specific force application. Differential adaptations emerge through configuration-specific emphasis: S1a prioritizes trunk rigidity via Progressive Plank to maintain streamlined postures during start entries and glide phases, whereas S1b leverages vertical jump training’s stretch-shortening cycle properties to amplify explosive start capabilities. These composite adaptations typify elite short-course front crawl swimming athletes combining robust foundational strength with refined hydrodynamic technique.

### 4.2 S2a and S2b

The S2a configuration identifies bench press, pull-ups, and vertical jump as core condition variables, with progressive plank and back squat classified as peripheral factors, while medicine ball chest throws and grip strength are considered non-determinant; this configuration accounts for approximately 48.7% of elite freestyle performance cases (3.4% uniquely explained). The S2b configuration similarly establishes bench press, pull-ups, and vertical jump as core elements, both configurations share an emphasis on upper-body pushing (bench press), vertical explosiveness (jump), and pulling strength (pull-ups), suggesting an integrated contribution to both start and stroke phases. Strength adaptations drive performance optimization: Kwok et al. (Yu Kwok et al., 2021) suggested that bench press enhances pectoralis major activation critical for stroke efficiency (Yu Kwok et al., 2021), while Born et al. (Born et al., 2020) suggested that vertical jump training improves start and turn acceleration via explosive triple extension (Born et al., 2020). Pull-ups ensure kinetic chain integrity through latissimus dorsi-centric contraction patterns, optimizing early-phase high-elbow catch mechanics complemented by jump-derived torso stiffness improving mid-pull stabilization. Start phases exhibit superior block velocity due to vertical jump engagement, while turns exploit ankle plantarflexion recoil for wall push-off enhancement. These configurations collectively represent athletes with refined stroke mechanics and start-turn proficiency, demonstrating that integrating vertical explosion (jump training) with horizontal thrust generation (bench press/pull-ups) creates a multidimensional power matrix surpassing traditional squat-dominated training paradigms, which is particularly advantageous for sprint-centric performance optimization.

### 4.3 S3

The S3 configuration emphasizes a broader base of full-body power, combining bench press, back squat, grip strength, and vertical jump. This configuration is unique in excluding pull-ups, indicating that some athletes may rely more on raw power than on pulling-specific strength. Progressive plank appears peripherally, while medicine ball throw is excluded. This configuration explains approximately 15% of elite freestyle performance cases, with 1.5% uniquely attributable to it. Athletes aligned with this configuration emphasize whole-body coordination, typically exhibiting higher kicking frequencies and superior starting capabilities. Mechanistically, bench press enhances horizontal propulsion by optimizing upper-limb thrust during the stroke phase, while back squat strengthens lower-body explosive power critical for start and turn performance. Vertical jump training improves vertical takeoff force, enhancing block start efficiency, and grip stability minimizes hydrokinetic hand slippage during propulsion. The exclusion of pull-ups (neglecting latissimus dorsi engagement) and medicine ball throws (suppressing trunk rotation dynamics) prioritizes direct power output over coordinated multi-planar movements. Progressive plank peripherally supports basic torso stability but does not dominate adaptability. This configuration may benefit short sprints where immediate force output is key, but its limited coverage suggests that omitting trunk and back synergy reduces its broader effectiveness. Athletes benefiting exclusively from S3 often possess inherent morphological or technical peculiarities, such as disproportionate limb leverage ratios or a neuromuscular preference for linear force transmission, that align with this simplified kinetic chain strategy. Consequently, S3 represents a specialized, supplementary training archetype, contrasting with the comprehensive S1 and S2 configurations favored by most elite performers due to their integrated power-coordination frameworks.

### 4.4 S4

In the S4 configuration, pull-ups, progressive core stabilization, and vertical jumps are identified as core condition variables, while bench press, back squat, and medicine ball chest throws are classified as marginally non-existent conditions. This configuration, though less frequent, may represent swimmers with highly developed posterior chains and core control, compensating for less traditional push-based strength. This indicates the S4’s highly limited applicability but efficacy in specialized cases. Its intrinsic mechanism prioritizes back pulling strength and core stability combined with vertical explosive power while disregarding traditional thrust-oriented training. It may cater to athletes with specific technical profiles—those relying predominantly on back musculature rather than pectoralis major for propulsion and emphasizing vertical jump-driven starts over horizontal thrust. The uniqueness of the 2.6% cases likely stems from athletes possessing distinct physiological traits: superior latissimus dorsi development with underdeveloped pectoral muscles, restricted shoulder mobility, exceptional core stability, or exceptional vertical takeoff velocity requirements during starts despite deficiencies in other physical attributes. These athletes may achieve performance through high-frequency technical execution, leveraging back-dominant mechanics at the cost of conventional force transmission pathways. The S4 configuration offers an alternative training paradigm for athletes with atypical physical characteristics, utilizing their anatomical peculiarities to circumvent traditional training limitations.

### 4.5 Commonality analysis across configurations

Across configurations S1 to S4, several recurring patterns emerge. Pull-ups appear in nearly all solutions, highlighting the importance of upper-body pulling strength, particularly for underwater propulsion. Vertical jump also features prominently, underlining the role of lower-limb explosiveness for starts and wall push-offs. Progressive plank is a frequent core or peripheral condition, reflecting the value of core stability for maintaining hydrodynamic position. In contrast, medicine ball throw appears only peripherally or is excluded entirely, suggesting its limited contribution in this cohort. These findings suggest that multiple high-performance profiles exist: some athletes excel via upper-body dominance, others through lower-body explosiveness, or through balanced core–limb coordination. Recognizing these configurations allows coaches to tailor training toward the individual athlete’s strengths while avoiding overgeneralized programs. Among the identified configurations, S1a/S1b and S2a/S2b should be prioritized in training practice, as they accounted for most high-performance cases and included core strength variables such as pull-ups, bench press, and vertical jump. These combinations reflect a balance between upper-body pulling, pushing, and lower-body explosive power, offering a robust foundation for improving sprint performance in adolescent swimmers. Coaches should particularly focus on these elements when designing generalized training protocols before individual profiles are fully assessed.

### 4.6 Conclusions and future directions

The study identified critical strength configurations that optimally enhance short-distance front crawl performance: Configurations S1a/S1b emphasize back squats, pull-up capacity, and handgrip strength as core factors, with peripheral variables influencing outcomes. Configurations S2a/S2b prioritize bench press performance and vertical jump metrics to optimize stroke efficiency and start/turn acceleration. Configuration S3 focuses on whole-body coordination dynamics, while Configuration S4 combines back muscle pulling strength, core stability, and vertical explosive power. Elite performance requires meeting strength baselines: simultaneous attainment of threshold levels in upper limb propulsion strength, core stability, and lower limb explosiveness.

Previous dry-land strength studies have typically applied linear correlation or regression methods to examine the relationship between isolated strength metrics and swimming performance. For instance, Amaro et al. (2017) and Crowley et al. (2017) reported significant associations between upper-body strength (e.g., bench press, pull-up) and sprint times. More recently, Zebura et al. (2023) found strong correlations between lower-body strength and 5–15 m starts velocities in elite swimmers. While these findings highlight important predictors, such approaches assume linear and additive effects. In contrast, our fsQCA results show that multiple, distinct configurations of strength variables can lead to excellent performance, revealing the presence of causal complexity and functional equivalence not captured by traditional models.

While current analyses primarily focus on strength training, unexamined technical factors may interact with strength adaptations. Additionally, the cross-sectional design of the study limits causal inference, as it does not allow us to determine whether strength improvements directly cause performance gains. Individual variability in training responsiveness underscores the necessity for personalized programming. Existing correlation-based findings should be supplemented with longitudinal studies to establish causal relationships between strength training and swimming performance. In addition, all dry-land strength tests in this study were performed within a single session with fixed 5-min rest intervals. Although this approach ensured procedural consistency, it may have introduced cumulative neuromuscular fatigue, particularly following maximal-effort tasks such as the bench press and back squat, which could have influenced later performance scores and affected the resulting configurations. Furthermore, swim trials were hand-timed, which may introduce minor measurement errors. While care was taken to standardize timing procedures, human reaction variability can affect the precision of short-distance swim trials. Another limitation is the lack of maturation control. Given the adolescent age range of the participants, differences in biological development (e.g., pubertal stage) could influence both strength levels and swimming efficiency, potentially confounding the relationship between strength and performance. Additionally, sex distribution was not recorded, which limits the ability to interpret strength and performance differences across groups. Given known sex-related differences in strength development during adolescence, this omission represents a further source of uncontrolled heterogeneity. Future research should expand to diverse age groups and swimming disciplines to validate configuration universality. The integration of strength development with technical skill refinement warrants exploration to design training protocols that concurrently enhance power output and movement economy. Moreover, the present study did not incorporate technical or psychological factors such as stroke mechanics, motor coordination, or mental readiness, all of which are known to influence swimming performance and interact with neuromuscular traits. The exclusion of relevant physical characteristics, such as flexibility and joint mobility, further limits the ecological validity of the findings. Including these variables would enhance the comprehensiveness of the strength–performance relationship. Future models should adopt a multidimensional approach that includes such variables to provide more holistic and actionable insights (Tedeschi, 2012).

These recommendations should, however, be interpreted considering the study’s methodological constraints. The sample, although sufficient for fsQCA, was regionally limited and may not fully represent broader adolescent swimmer populations. Future research should include longitudinal tracking of strength training adaptations and their impact on swimming performance to strengthen causal interpretation. Validation of the current findings across other swimming events (e.g., backstroke, butterfly), age groups, and sex categories is also warranted to assess generalizability. Furthermore, combining fsQCA with supervised machine learning models (e.g., decision trees, random forests) could support the development of practical diagnostic tools to classify athletes into strength-performance configurations and personalize training interventions. Finally, although this study identified multiple configurations of strength traits associated with high-level performance, their overlapping nature may limit immediate practical application. Without diagnostic tools to classify individual athletes into specific configurations, the translation into personalized training plans remains a conceptual proposition. In addition, the configurational model was not cross-validated on an independent sample, which limits its generalizability. Future research should aim to replicate these findings in separate cohorts and explore decision trees or supervised models to facilitate configuration-based training guidance.

## Data Availability

The original contributions presented in the study are included in the article/supplementary material, further inquiries can be directed to the corresponding author.
